# Supplementary material for: Association between platelet distribution width and prognosis in patients with heart failure
Source: PLoS One. 2020 Dec 29;15(12):e0244608. doi: 10.1371/journal.pone.0244608 (PMC7771660; doi:10.1371/journal.pone.0244608)
Supplement: S1 Table — OR, odds ratio; CI confidence interval; CAD, coronary artery disease; CKD, chronic kidney disease; RAS, renin-angiotensin system; Log-BNP, log-transformed B-type natriuretic peptide. *Adjusted for age, sex, and factors which had P values of < 0.05 in the unadjusted model. (DOCX) [file pone.0244608.s001.docx]

**S1 Table. Logistic regression analysis for the 3^rd^ tertile (n = 543/1,746)**

|  | **Unadjusted** | |  | **Adjusted *** | |
| --- | --- | --- | --- | --- | --- |
|  | **OR (95% CI)** | **P value** |  | **OR (95% CI)** | **P value** |
| Age | 1.010 (1.003–1.017) | 0.008 |  | 1.005 (0.997–1.013) | 0.218 |
| Male sex | 1.072 (0.872–1.319) | 0.510 |  | 1.139 (0.908–1.427) | 0.260 |
| Hypertension | 0.811 (0.655–1.003) | 0.054 |  | – | – |
| Diabetes mellitus | 1.371 (1.114–1.686) | 0.003 |  | 1.352 (1.084–1.687) | 0.008 |
| Dyslipidemia | 0.986 (0.791–1.229) | 0.901 |  | – | – |
| Hyperuricemia | 1.295 (1.053–1.595) | 0.015 |  | 1.007 (0.790–1.282) | 0.957 |
| Anemia | 1.370 (1.118–1.679) | 0.002 |  | 1.275 (1.021–1.593) | 0.032 |
| CAD | 0.939(0.754–1.169) | 0.574 |  | – | – |
| CKD | 1.399 (1.140–1.716) | 0.001 |  | 1.160 (0.908–1.481) | 0.235 |
| RAS inhibitors | 0.865 (0.693–1.080) | 0.201 |  | – | – |
| Antiplatelet agents | 0.926 (0.756–1.135) | 0.460 |  | – | – |
| Log-BNP | 1.313 (1.102–1.563) | 0.002 |  | 1.160 (0.958–1.404) | 0.129 |

OR, odds ratio; CI confidence interval; CAD, coronary artery disease; CKD, chronic kidney disease; RAS, renin-angiotensin system; Log-BNP, log-transformed B-type natriuretic peptide.

*Adjusted for age, sex, and factors which had P values of < 0.05 in the unadjusted model.
