# Supplementary material for: Association between platelet distribution width and prognosis in patients with heart failure
Source: PLoS One. 2020 Dec 29;15(12):e0244608. doi: 10.1371/journal.pone.0244608 (PMC7771660; doi:10.1371/journal.pone.0244608)
Supplement: Attachment — Submitted filename: Response to Reviewers.docx [file pone.0244608.s003.docx]

**Reply to the Academic Editor**

Thank you for your helpful comments on our manuscript (PONE-D-20-22248), which we have revised accordingly.

**Academic Editor’s Comment 1:**

**Thank you for submitting your manuscript to PLOS ONE. After careful consideration, we feel that it has merit but does not fully meet PLOS ONE’s publication criteria as it currently stands. Therefore, we invite you to submit a revised version of the manuscript that addresses the points raised during the review process.**

**Please note that the manuscript has important limitations, which make it not acceptable in its present form. Specifically, the question arises, as also addressed by the reviewer, why additional prognostic markers are needed in heart failure. in fact, I do not see any reasons unless a novel marker is extremely outstanding in this regard, which is not the case. There are already so many and in clinical practice, the impact even of the most established ones (e.g. NT pro-BNP / BNP) is unclear. Therefore, the findings of a new prognostic marker is scientifically not interesting, unless the study provides novel pathophysiological insight. Unfortunately, this is hardly addressed in the manuscript, which is a very important limitation of the manuscript.**　 **Additional analyses addressing potential pathophysiological mechanisms may help in this regard. The authors only show differences in baseline characteristics between tertiles of PWD, but do not address factors potentially influencing PWD. This could help to estimate the meaning and as a consequence, also the potential value of PWD. This needs to be properly addressed.**

Author’s Response 1:

We appreciate the important comments from the editor and reviewers. We agree that natriuretic peptides (NPs) are established markers in the management of HF patients. However, aside from NPs, novel biomarkers may improve the understanding of the complex disease process of HF, and possibly personalized care for those affected through better individual phenotyping or understanding pathophysiologic pathways (e.g. myocardial stretch/stress, cardiac extracellular matrix remodeling, cardiomyocyte injury/death, oxidative stress, inflammation, neurohumoral activation, and renal dysfunction). Thus, a multi-biomarker approach for the improvement in management of HF and the personalization of care have been considered. In addition, the advantage of PDW is that PDW is reported automatically and routinely as part of a complete blood count without any additional equipment or techniques. In other words, this study found a new value in the existing test. We discussed the utility of a multi-biomarker approach other than NPs, and clearly mentioned the strength of PDW in the Discussion section (page 11, lines 170-172). Furthermore, we performed the logistic regression analysis (S1 Table) in order to determine the factors potentially influencing PDW in accordance with the editor’s comments. We mentioned the implication of PDW in the Discussion section (page 12, line 198 – page 13, line 216). From this study, the clinical implication of PDW should be further clarified. We mentioned this in the Limitation section (page 13, lines 227-228).

Copy of the revised text

“These findings are pivotal for clinical practice because complete blood count is performed in almost all patients with HF, and PDW is reported routinely and automatically.” (page 11, lines 170-172)

“Although there have been numerous biomarkers reported in patients with HF, accurate risk stratification remains challenging. Natriuretic peptide (NP) is widely used to support diagnosis, prognostication, and management of patients with HF, but NPs come with limitations, including vulnerability to the presence of obesity, atrial fibrillation, and renal dysfunction, for example. Aside from NPs, novel biomarkers may improve the understanding of the complex disease process of HF, and possibly personalize care for those affected through better individual phenotyping or understanding pathophysiologic pathways (e.g. myocardial stretch/stress, cardiac extracellular matrix remodeling, cardiomyocyte injury/death, oxidative stress, inflammation, neurohumoral activation, and renal dysfunction). In these regards, it is reasonable to expect the application of a multibiomarker approach for the improvement in management of HF and the personalization of care. From the results of our study, the presence of diabetes mellitus and anemia, but not B-type natriuretic peptide, was associated with elevated levels of PDW. This suggested that the underlying mechanisms of high PDW were different from left ventricular stretch or diastolic wall stress. Instead, the prothrombotic state due to insulin resistance and insulin deficiency may have contributed to increasing platelet reactivity. Anemia is associated with myocardial remodeling, inflammation, and volume overload in patients with HF. However, the link between anemia and PDW has been unclear. Thus, PDW is useful for multifaceted assessment of the pathophysiology of HF.” (page 12, line 198 – page 13, line 216)

“The underlying mechanisms influencing the levels of PDW need more investigation.” (page 13, lines 227-228)

**Editor’s Comment 2:**

**In addition, there are minor issues to solve. Thus, the authors should avoid to repeat results in the text that are already given in tables or figures.**

Author’s Response 2:

We have accordingly excluded the repeated results from the text which were already given in the Tables or Figures.

**Editor’s Comment 3:**

**The authors need to make clear, why they did not include more co-morbidities and also not including them in the multivariable model. Moreover, the authors do not consider potential dependencies between variables in multivariable models. The language of the manuscript needs to be improved as well. Take together, a substantial change of the manuscript and the analysis is required before the manuscript may be considered for publication. I would like to leave it to the authors if they are willing to perform such substantial changes. If not, the manuscript will be rejected, however.**

Author’s Response 3:

Thank you for your comment. According to your suggestions, we have extensively revised the manuscript. We included more comorbidities (i.e. diabetes mellitus, dyslipidemia, hyperuricemia, atrial fibrillation, peripheral artery disease, chronic kidney disease, chronic obstructive pulmonary disease) in the revised Table 1 and input them into a multivariate Cox proportional hazard analysis in the revised Table 2. In order to avoid arbitrary selection bias, we adjusted the impact of PDW by age, sex, and factors that were statistically different among the groups. We have mentioned this point in the Statistical Methods (page 5, lines 98-101). After the revision, the prognostic impact of PDW was essentially consistent with our previous analysis. The manuscript was then checked by native English-speaking scientific editors.

Copy of the revised text

“We assessed PDW as a predictor of the primary endpoints using the Cox proportional hazard analysis. Hazard ratio (HR) and 95% confidence interval (CI) were further adjusted for age, sex, and variables that were significantly different among the groups.” (page 5, lines 98-101)

**Editor’s Comment 4:**

**If applicable, we recommend that you deposit your laboratory protocols in protocols.io to enhance the reproducibility of your results. Protocols.io assigns your protocol its own identifier (DOI) so that it can be cited independently in the future.**

Author’s Response 4:

Since this was an observational clinical research based on daily clinical care, and PDW was routinely measured using an automated hematology analyzer in our hospital, we did not use specific experimental materials. In these regards, we could not present laboratory protocols, instead we clearly described the equipment used in the Methods. (page 5, lines 88-90)

Copy of the revised text

“PDW was analyzed from anticoagulated blood (ethylenediaminetetraacetic acid bulb) using an automated hematology analyzer, UniCel DxH 800 (BECKMAN COULTER, Brea, CA, USA) within 1 week prior to discharge in a stable condition.” (page 5, lines 88-90)

Thank you again for your helpful suggestions for our study.

**Reply to the Reviewer**

Thank you for your helpful comments on our manuscript (PONE-D-20-22248), which we have revised accordingly.

**Reviewer’s Comment 1:**

**Line 29: Age was oldest. should be rewritten. This should also be modified further along in the manuscript.**

Author’s Response 1:

We have revised the manuscript accordingly (page 2, lines 24-26; page 6, lines 118-120).

Copy of the revised text

“The 3^rd^ tertile showed the highest age and levels of B-type natriuretic peptide compared to other tertiles (1^st^, 2^nd^, and 3^rd^ tertiles; age, 69.0, 68.0, and 70.0 years old, P = 0.038; B-type natriuretic peptide, 235.2, 171.9, and 241.0 pg/mL, P < 0.001).” (page 2, lines 24-26)

“There were U-shaped associations between PDW tertiles and several important factors such as age, hypertension, diabetes mellitus, coronary artery disease, and levels of B-type natriuretic peptide.” (page 6, lines 118-120)

**Reviewer’s Comment 2:**

**Introduction: Although the authors mention the CBC, they do not mention the value of hematocrit which has been studied as a prognostic marker in HF.**

Author’s Response 2:

We appreciate the helpful comment. We have mentioned the prognostic impact of hematocrit in the revised manuscript (page 3, lines 41-42).

Copy of the revised text

“However, the usefulness of complete blood count other than hemoglobin or hematocrit in predicting prognosis has yet to be fully evaluated.” (page 3, lines 41-42)

**Reviewer’s Comment 3:**

**Table 1: although the prevalence of AFib in this patient population was close to 40%, anticoagulation was used in 54-60% of patients. Could the authors explain the discrepancy?**

Author’s Response 3:

In the whole study population, a total of 434 patients were prescribed anticoagulants for reasons other than atrial fibrillation. Among them, 171 (39.4%) patients had undergone cardiac surgery (CABG, AVR, MVR, etc.). It is not rare for patients with heart failure to obtain anticoagulation due to various reasons such as post-operatic state, intracardiac thrombosis, venous thromboembolism, etc. We mentioned the discrepancy in the Limitations section (page 13, line 230-232).

Copy of the revised text

“There were some patients receiving anticoagulants for reasons other than atrial fibrillation (e.g. post-operatic state). However, the leading cause of anticoagulant therapy was not taken into consideration in this study.” (page 13, line 230-232)

**Reviewer’s Comment 4:**

**Table 1: LVEF was around 50%. Does this actually reflect the population normally admitted to hospital with acute decompensated HF? Previous studies of all hospitalizations for HF usually enroll patients with a mean EF slightly lower.**

Author’s Response 4:

Thank you for making this important point. Recent studies have demonstrated that heart failure with preserved (50% or more) ejection fraction represents up to half of the whole heart failure population (Michel Komajda, Eur Heart J. 2014; Dunlay, S.M, Nat Rev Cardiol. 2017). As you mentioned, previous studies sometimes focused on heart failure patients with reduced ejection fraction due to study protocols. In this study, heart failure was diagnosed on the basis of current guidelines, rather than ejection fraction.

**Reviewer’s Comment 5:**

**Line 177: The authors does raise an important question regarding the discrepancy of some of their findings. Additional work is needed to better understand this finding.**

Author’s Response 5:

Thank you for pointing out these important unsolved issues. PDW is affected not only by atherosclerosis but also by hyperglycemia, cancer, infection, aplastic anemia, idiopathic thrombocytopenic purpura, etc. We fully agree that additional work is needed to better understand the discrepancy of some of our findings. This study should be considered as preliminary since it was performed in a single center with a relatively small number of participants. We have mentioned this point in the Limitations section (page 13, lines 227-228).

Copy of the revised text

“The underlying mechanisms influencing the levels of PDW need more investigation.” (page 13, lines 227-228)

**Reviewer’s Comment 6:**

**Overall, although these findings are interesting, I am not convinced that we need an additional prognostic marker for HF when strong biomarkers such as BNP and NT proBNP are most often available.**

Author’s Response 6:

We agree that natriuretic peptides (NPs) are established markers in the management of HF patients. However, aside from NPs, novel biomarkers may improve the understanding of the complex disease process of HF, and possibly personalize care for those affected through better individual phenotyping or understanding pathophysiologic pathways (e.g. myocardial stretch/stress, cardiac extracellular matrix remodeling, cardiomyocyte injury/death, oxidative stress, inflammation, neurohumoral activation, and renal dysfunction). Thus, a multi-biomarker approach for the improvement in management of HF and the personalization of care have been considered. In addition, the strength of PDW is that it does not require additional equipment or techniques and is reported automatically and routinely in daily practice. We discussed the utility of a multi-biomarker approach other than NPs, and clearly mentioned the strength of PDW in the Discussion section (page 11, lines 170-172). In addition, we added a logistic regression analysis (S1 Table) in order to determine the factors associated with elevated levels of PDW. We found that the presence of diabetes mellitus and anemia, but not B-type natriuretic peptide, was associated with high PDW. This result suggested that the underlying mechanisms of elevated levels of PDW were different from left ventricular stretch or diastolic wall stretch. Thus, evaluating the levels of PDW is useful for the multifaceted understanding of the pathophysiology of heart failure. We mentioned this in the Discussion section (page 12, line 198 - page 13, line 216).

Copy of the revised text

“These findings are pivotal for clinical practice because complete blood count is performed in almost all patients with HF, and PDW is reported routinely and automatically.” (page 11, lines 170-172)

“Although there have been numerous biomarkers reported in patients with HF, accurate risk stratification remains challenging. Natriuretic peptide (NP) is widely used to support diagnosis, prognostication, and management of patients with HF, but NPs come with limitations, including vulnerability to the presence of obesity, atrial fibrillation, and renal dysfunction, for example. Aside from NPs, novel biomarkers may improve the understanding of the complex disease process of HF, and possibly personalize care for those affected through better individual phenotyping or understanding pathophysiologic pathways (e.g. myocardial stretch/stress, cardiac extracellular matrix remodeling, cardiomyocyte injury/death, oxidative stress, inflammation, neurohumoral activation, and renal dysfunction). In these regards, it is reasonable to expect the application of a multibiomarker approach for the improvement in management of HF and the personalization of care. From the results of our study, the presence of diabetes mellitus and anemia, but not B-type natriuretic peptide, was associated with elevated levels of PDW. This suggested that the underlying mechanisms of high PDW were different from left ventricular stretch or diastolic wall stress. Instead, the prothrombotic state due to insulin resistance and insulin deficiency may have contributed to increasing platelet reactivity. Anemia is associated with myocardial remodeling, inflammation, and volume overload in patients with HF. However, the link between anemia and PDW has been unclear. Thus, PDW is useful for multifaceted assessment of the pathophysiology of HF.” (page 12, line 198 - page 13, line 216)

Thank you again for your helpful suggestions for our study.
