# Supplementary material for: Association between platelet distribution width and prognosis in patients with heart failure
Source: PLoS One. 2020 Dec 29;15(12):e0244608. doi: 10.1371/journal.pone.0244608 (PMC7771660; doi:10.1371/journal.pone.0244608)
Supplement: Attachment — Submitted filename: Response to Reviewers.docx [file pone.0244608.s004.docx]

**Reply to the Editor**

Thank you for your helpful comments on our manuscript (PONE-D-20-22248R1), which we have revised accordingly.

**Academic Editor’s Comment 1:**

**I would like to thank the authors for addressing most of the raised points. However, there is still a remaining issue that may be improved. Although I know that the pathophysiology of PDW is not yet investigated in much detail, the authors should address the pathophysiological part of finding a new biomarker in general and specifically for PDW more in depth. As mentioned in the previous comments, simply finding a new marker is not of much value, even if such a marker is easy to determine. It is not only (NT-pro)BNP which is a good prognostic marker, but there are so many other markers (including biomarkers) found that are related to outcome that a new one must be exceptionally good in predicting outcome (which is not the case here) or help the reader to understand a (new) pathophysiological process.**

**Author’s Response 1:**

We appreciate this opportunity to reconsider our findings. Indeed, the underlying mechanisms of increased PDW have not been fully elucidated. However, there have been increasing knowledge which support the speculation about the pathophysiological process and clinical implications of PDW. We discussed these aspects in depth, and revised the Introduction (page 3, lines 52-56) and the Discussion (page 12, line 195 – page 13, line 215).

Copy of the revised text

“Moreover, levels of PDW predicts the occurrence of cardiac death, infarction recurrence, and another revascularization in patients with acute myocardial infarction.[7, 15] In addition to routine clinical laboratory tests, new biomarkers reflecting various pathophysiological aspects can be useful adjuncts for the diagnosis, prognosis prediction, and treatment of HF. [1, 2]” (page 3, lines 52-56)

“From the results of our study, the presence of diabetes mellitus and anemia, but not B-type natriuretic peptide, was associated with elevated levels of PDW. This suggested that the underlying mechanisms of high PDW were different from left ventricular stretch or diastolic wall stress [38, 39]. Levels of PDW show a positive correlation with hemoglobin A1c [40]. Patients with diabetes mellitus, particularly those with microvascular complications, show increased levels of PDW [41]. Previous studies suggested that the prothrombotic state due to insulin resistance and insulin deficiency may have contributed to increasing platelet reactivity [41, 42]. Patients with diabetes mellitus demonstrate increased platelet activation and increased release of prothrombotic and proinflammatory agents due to systematic inflammation, oxidative stress, impaired calcium metabolism, decreased bioavailability of nitric oxide, increased phosphorylation and glycosylation of cellular proteins [43]. More active platelets have more prothrombic contents, such as thromboxane A2, thromboxane B2, platelet factor 4, serotonin, and platelet-derived growth factor [44]. Thus, HF patients with increased PDW are suspected to have these underlying pathophysiologies. On the other hand, anemia is associated with myocardial remodeling, inflammation, and volume overload in patients with HF [45]. However, the link between anemia and PDW has been unclear. Levels of PDW depend on whether platelets are hypoproductive such as aplastic anemia or hyperdestructive [46]. The clinical implications of PDW in anemia should be investigated by further studies because the leading cause of anemia was not recorded in this study. Thus, PDW is useful for multifaceted assessment of the pathophysiology of HF.” (page 12, line 195 – page 13, line 215)

Thank you again for your helpful suggestions for our study.
